# Targeting the Epidermal Growth Factor Receptor in Epithelial Ovarian Cancer: Current Knowledge and Future Challenges

**DOI:** 10.1155/2010/568938

**Published:** 2009-11-19

**Authors:** Doris R. Siwak, Mark Carey, Bryan T. Hennessy, Catherine T. Nguyen, Mollianne J. McGahren Murray, Laura Nolden, Gordon B. Mills

**Affiliations:** ^1^Department of Systems Biology, The University of Texas M. D. Anderson Cancer Center, 1515 Holcombe Boulevard, Houston, TX 77030, USA; ^2^Department of Gynecologic Medical Oncology, The University of Texas M. D. Anderson Cancer Center, 1515 Holcombe Boulevard, Houston, TX 77030, USA

## Abstract

The epidermal growth factor receptor is overexpressed in up to 60% of ovarian epithelial malignancies. EGFR regulates complex cellular events due to the large number of ligands, dimerization partners, and diverse signaling pathways engaged. In ovarian cancer, EGFR activation is associated with increased malignant tumor phenotype and poorer patient outcome. However, unlike some other EGFR-positive solid tumors, treatment of ovarian tumors with anti-EGFR agents has induced minimal response. While the amount of information regarding EGFR-mediated signaling is considerable, current data provides little insight for the lack of efficacy of anti-EGFR agents in ovarian cancer. More comprehensive, systematic, and well-defined approaches are needed to dissect the roles that EGFR plays in the complex signaling processes in ovarian cancer as well as to identify biomarkers that can accurately predict sensitivity toward EGFR-targeted therapeutic agents. This new knowledge could facilitate the development of rational combinatorial therapies to sensitize tumor cells toward EGFR-targeted therapies.

## 1. Introduction

Epithelial ovarian cancer, defined as cancers arising either from the mesothelial lining of the ovaries (either from the epithelial surface lining or cortical ovarian cysts formed by invaginations of the surface epithelium) or from the fallopian tube epithelium [1], accounts for 90% of ovarian malignancies [2]. Epithelial ovarian cancers are further divided into 5 histologic subtypes: serous, endometrioid, mucinous, clear cell, and undifferentiated. Aberrant epidermal growth factor receptor (EGFR) expression is detected in up to 60% of ovarian cancers and occurs in all histologic subtypes [3, 4]. Further, aberrant EGFR expression is associated with poor outcome of ovarian cancer patients [5, 6]. In this article, we review the EGFR family, the role of EGFR in ovarian cancer, and the methods used to determine this role. We also summarize the results of anti-EGFR therapies in ovarian cancer clinical trials and discuss challenges and future work in effective treatments utilizing anti-EGFR therapies in ovarian cancer, focusing on epithelial ovarian cancer whenever possible.

### 1.1. The Epidermal Growth Factor Receptor Family

**Figure 1:**
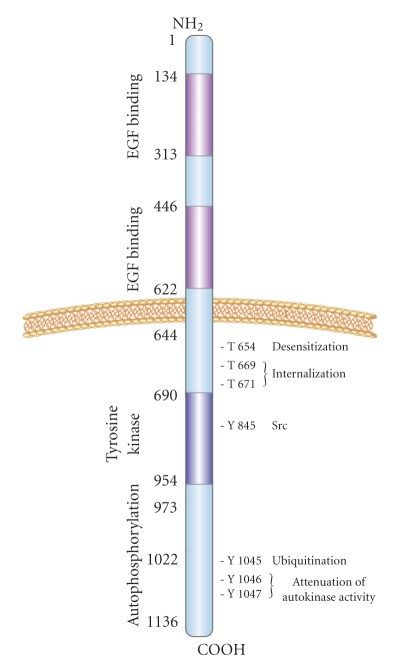
Structure of EGFR. EGFR consists of extracellular, transmembrane, and intracellular domains. The extracellular domain is the least conserved domain among the EGFR family members and consists of 4 subdomains—two ligand-binding domains and two receptor dimerization domains, which are cysteine-rich (reviewed in [12]). The transmembrane domain, which spans the cell membrane, is hydrophobic. The cytoplasmic tail of the EGFR family is highly conserved and contains the tyrosine kinase domain. Activation of EGFR family members leads to autophosphorylation of the tyrosine residues in the cytoplasmic tail. The phosphorylated tyrosine residues become docking sites for proteins with SRC homology 2 and phosphotyrosine binding domains, which transduce the signals downstream. EGFR phosphorylation at selected residues and their functional outcomes are indicted in the diagram. T: threonine; Y: tyrosine.

The EGFR family (also known as the HER or ERBB family) consists of 4 members: EGFR, HER2, HER3, and HER4 (alternately known as ERBB1–4). Structurally, the EGFR family consists of an extracellular ligand binding domain, a single transmembrane-spanning region, and an intracellular region containing the kinase domain (Figure 1; reviewed in [7–10]). In humans, more than 30 ligands have been identified that bind to the EGFR family, including EGF and EGF-like ligands, transforming growth factor (TGF)-*α*, and heregulins (HRGs, also known as neuregulins) [11].

EGFR is activated upon ligand binding, which results in a conformational change in the extracellular domain, leading to homo- or heterodimerization with another EGFR family member. The EGFR binding partner appears to depend on several properties, including the proportion of EGFR family members in the membrane, type and proportion of ligand (reviewed in [10, 13]), and cell lineage likely reflected in the expression of additional members of the signaling complex (see below). Strikingly, HER2 is the preferred binding partner for all EGFR family members [14], while HER3 is an obligatory partner [15], being inactive on its own or as a homodimer as it lacks intrinsic kinase activity due to mutation of critical amino acids in the kinase domain [16, 17]. This combination has lead to the suggestion by Yarden and colleagues that HER2 and HER3 are “deaf and dumb” members of the EGFR family, functioning in normal physiology as part of signaling complexes with other EGFR family members [18].

**Figure 2:**
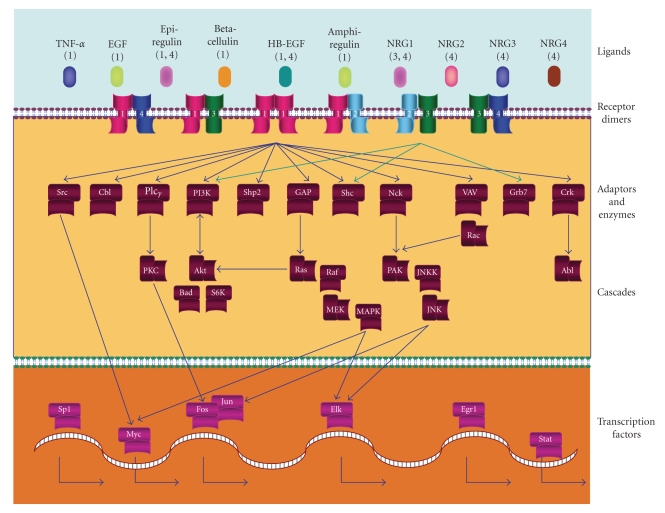
Selected representation of canonical EGFR family signaling pathways. The EGFR family consists of 4 members: EGFR, HER2, HER3, and HER4 (indicated by numbers 1–4 in the diagram). EGFR family ligands include EGF-and EGF-like ligands, transforming growth factor (TGF)-*α* and heregulins (HRGs, also known as neuregulins, NRGs). As indicated by the numbers in parentheses beneath the ligands, each ligand binds preferentially to a particular EGFR family member. HER2, while lacking any known ligand, is the preferred binding partner of for all EGFR family members. HER3 lacks intrinsic kinase activity due to mutation of critical amino acids in the kinase domain; therefore, it is inactive on its own or as a homodimer. Transduction of EGFR signals occurs through intracellular adaptor proteins, which transmit signals through cascades such as the RAS/RAF/MEK/mitogen-activated protein kinase (MAPK) and phosphatidylinositol 3′-kinase (PI3K)/AKT cascades. The downstream proteins in these signaling cascades can shuttle from the cytoplasm to the nucleus, where they signal to transcription factors and their complexes such as MYC, ELK, and FOS/JUN. Signal transduction through the EGFR family to downstream pathways and cascades controls diverse cellular responses such as proliferation, differentiation, cell motility, and survival as well as tumorigenesis. Figure adapted from [13]. Abbreviations: PLC*γ*: Phospholipase C*γ*; SHP2: SRC homology phosphatase 2; GAP: GTPase activating protein; SHC: SRC homology 2 domain and collagen-containing protein; PKC: Protein kinase C; MEK: MAPK/ERK kinase; PAK: P21-activated kinase; JNKK: JNK kinase; JNK: JUN N-terminal kinase; EGR1: Early growth response protein 1; STAT: Signal transducer and activator of transcription.

Activation of the EGFR family members results in transduction of EGFR signals, via intracellular cascades, such as mitogen-activated protein kinases (MAPKs), and AKT (also known as protein kinase B), resulting in perturbation of multiple cellular responses including proliferation, differentiation, cell motility, and survival (reviewed in [9, 19]). A summary of selected EGFR family pathways is shown in Figure 2. 

 The EGFR family members can also be activated by other signaling proteins independent of addition of exogenous EGFR ligands. These include other receptor tyrosine kinases (RTKs) such as insulin-like growth factor-1 receptor (IGF-1R) (reviewed in [20, 21]) and tyrosine kinase receptor B (TRKB, [22]) as well as other types of receptors such as G protein-coupled receptors (GPCRs) (reviewed in [23]), the leptin receptor [24], and adhesion proteins such as E-cadherin (reviewed in [25]) and integrins (reviewed in [26]). While the details of EGFR transactivation upon crosstalk are not yet fully elucidated, transactivation has been shown to occur by a variety of mechanisms. For example, there is evidence that EGFR can be transactivated by IGF-1R by direct binding [27]. Additionally, EGFR transactivation by GPCR has been shown to occur intracellularly, such as by activation of SRC upon GPCR stimulation (e.g., [28]), as well as extracellularly, such as by GPCR activation by gastrin releasing peptide [29]. This induces the formation of a GPCR complex containing SRC, Phosphatidylinositol 3′-kinase (PI3K), PDK1, and TNF-*α* converting enzyme (TACE), resulting in activation and translocation of TACE to the membrane where it releases the EGFR ligand amphiregulin, resulting in subsequent EGFR activation [29]. Lysophosphatidic acid (LPA)-GPCR-induced ectodomain shedding of pro Heparin Binding-EGF also activates EGFR [30]. LPA-mediated signaling is of particular importance in ovarian cancer as abnormalities in LPA metabolism and function likely contribute to initiation and progression of ovarian cancer [31–33]. Additionally, TRKB may also play a role in ovarian cancer as its activation has been shown to enhance migration and proliferation and suppress anoikis in human ovarian cancer cells [22, 34].

### 1.2. EGFR in Ovarian Cancer

The *EGFR* gene, located on chromosome 7p12, is amplified in ovarian cancer in approximately 4%–22% of cases [3, 6, 35, 36], including about 13% in epithelial ovarian cancers [35]. Activating *EGFR* mutations, as determined by sequence analyses of potential activating mutation sites in the catalytic domain, is rare in ovarian cancer, with a frequency of 4% or less [6, 35, 37]. The constitutively active mutant *EGFRvIII*, while reported earlier to be detected in 73% (24/32) of ovarian cancers [38], was not detected in subsequent and more extensive studies examining serous [6] or various types of ovarian cancers [39]. Overexpression of the EGFR protein has been detected in 9%–62% of human ovarian cancers [6, 36, 40, 41]; the differences in frequencies from these studies likely reflect utilization of different antibodies and cutoffs for overexpression. *EGFR* gene amplification or protein overexpression occurs across all epithelial ovarian cancer histotypes [3, 4]. Increased EGFR expression has been associated with high tumor grade [3, 5, 6], high cell proliferation index [6], aberrant P53 expression [6], and poor patient outcome [5, 6].

One of the first studies implicating the EGFR pathway in ovarian cancer was the detection of TGF-*α* in human ovarian cancer effusions as determined by radioimmunoassay [42]. TGF-*α* was also shown to increase proliferation as measured by [^3^H]thymidine incorporation [43] as well as increase levels of the tumor markers cancer antigen-125 and tissue polypeptide antigen [44] in EGFR-positive primary human serous ovarian cancer cells. In the human ovarian adenocarcinoma cell line OMC-3, TGF-*α* induced migration and invasion as well as gelatinolytic, caseinolytic, and plasmin activity in a dose-dependent manner [45].

While initial studies suggested that EGF, due to the inability to detect transcripts in Northern blotting, might not play a significant role in ovarian cancer [43], subsequent studies indicated that exogenous EGF can also induce effects associated with transformation. Like TGF-*α*, treatment of OMC-3 cells with EGF induced cell migration and invasion and degradation of extracellular matrix components [45]. Additionally, human ovarian cancer cell lines treated with EGF showed significant increases in expression of proteins associated with invasion (urokinase plasminogen activator and its receptor, and plasminogen activator inhibitor-1 [46]). EGF can also affect pathways associated with angiogenesis, as EGF stimulation of the human ovarian adenocarcinoma cell line OVCAR-3 leads to increased H_2_O_2_ levels, which in turn activates the AKT-P70S6K pathway and increases vascular endothelial growth factor transcription through hypoxia-inducible factor-1*α* expression [47].

While earlier studies focused on EGFR ligands in ovarian cancer, emerging studies examined the mechanism of EGFR activation itself. For example, Campiglio et al. detailed the activation characteristics of the EGFR family members upon addition of EGF or HRG in human ovarian cancer cell lines containing different levels of EGFR family proteins [48]. In this report, they show that the pattern of EGFR family activation in human ovarian cancer cells appears to be distinct from that of human breast cancer cell lines; while EGFR and HER2 were consistently activated upon EGF treatment, HER3 and HER4 activation depended upon the relative abundance of each receptor in ovarian cancer cells. Additionally, HER3 activation could occur independently of HER2 [48]. This complex pattern of EGFR family activation could in part explain the poor rate of response to EGFR inhibition in ovarian cancer.

Further elucidation of the effects of EGFR signaling in ovarian cancer comes from inhibition of EGFR in cultured human ovarian cancer cells. For example, treatment of the human ovarian serous epithelial cancer cell line OVCA420 with the anti-EGFR murine monoclonal antibody (mAb) C225 resulted in decreased levels of cell cycle progression-associated proteins Cyclin-dependent kinase (CDK) 2, CDK4, and CDK6 and increased expression of the cell cycle-inhibiting protein P27^Kip1^, along with increased association of P27^Kip1^ with the CDKs [49]. Additionally, modulation of other cell cycle proteins was observed, including decreased expression and phosphorylation of the CDK substrates RB and P130 and decreased protein levels of cyclin A. Modulation of these proteins upon C225 treatment was associated with an increase in the proportion of cells in the G1 phase of the cell cycle. The effects observed upon EGFR inhibition were enhanced upon combined treatment of human ovarian cancer cells with the anti-HER2 murine mAb 4D5 [49].

As transactivation pathways in various cell systems have been delineated, so have the pathways associated with EGFR family activation in ovarian cancer. For example, Vacca et al. have provided evidence that the GPCR ligand, endothelin (ET)-1, can activate EGFR in the human ovarian cancer cell line OVCA 433 [50]. ET-1 has been observed to play a role in mitogenic autocrine loops in various cultured cell types including human ovarian cancer [51, 52] and is proposed to contribute to tumor growth in vivo [53]. ET-1 treatment increased phosphorylation of EGFR and its downstream proteins SRC homology 2 domain and collagen-containing protein (SHC) and ERK2 as well as increased SHC-GRB2 association [50]. These effects were reversed upon pretreatment of OVCA 433 cells with the EGFR inhibitor tyrphostin AG1478 as well as the ET_A_-specific antagonist BQ-123 [50].

More recent studies have found additional signaling molecules or pathways that contribute to EGFR-mediated malignant phenotype in human ovarian cancer cell lines, including EGFR-interleukin-6 crosstalk through Janus kinase 2/Signal transducer and activator of transcription 3 signaling to mediate epithelial-mesenchymal transition [54], coactivation of Src/EGFR and axin/glycogen synthase kinase (GSK)-3*β* pathways and induction of invasion by *β*-arrestin activation of the ET-A receptor [55], and Src/EGFR transactivation, cyclooxygenase-2 expression, and cell migration upon LPA2 stimulation in CAOV-3 cells [56].

## 2. Disease Models, Knockouts, and Assays for EGFR in Ovarian Cancer

In addition to the studies alluded to above in determining the effects of molecular modulations of EGFR and its biochemical and biological effects, several other approaches for studying EGFR have been used; these are summarized in Table 1. As EGFR is an extracellular signaling protein, the assays most commonly used in examining EGFR in human ovarian cancer cell lines or tissues involve methods that directly or indirectly measure EGFR activity. Assays include methods for detecting increased levels of the *EGFR* gene (e.g., fluorescence in situ hybridization) or protein (e.g., immunohistochemistry, Western blotting) as well as expression of activating *EGFR* mutations (e.g., polymerase chain reaction + sequencing) or measurement of EGFR protein activity (e.g., Western blotting of EGFR phosphorylation sites, in vitro kinase assays).

To determine the effects of EGFR activation or inhibition in tumor formation, human ovarian tumor cells are most frequently implanted heterotopically (subcutaneously) in immunocompromised mice (Table 1). No reports of “true orthotopic” implantation such as in the ovarian bursa of mice have been found in EGFR studies in ovarian cancer, presumably due to the complex and labor-intensive nature of these procedures, while a few reports of “semiorthotopic” implantations via intraperitoneal (IP) injection were identified. While IP tumor implantation offers a model potentially more reflective of advanced ovarian cancer in the patient than subcutaneous injection [57], the difficulty in measuring tumor volume in intact mice has precluded its widespread use in anti-EGFR drug studies.

In addition to implantation of human tissues or cells via xenografts, animal models utilizing other methods of tumor formation have been used to study ovarian cancer. (For comprehensive reviews on animal tumor models, see [58–61].) Most of these animal models utilize mice, and the methods used to induce tumor formation include (1) exposure to radiation (e.g., [62]) or chemicals (carcinogens or hormones) introduced at or near the ovary (e.g., [63]), (2) syngeneic models in which spontaneously transformed murine ovarian epithelial cells are transplanted into immunocompetent mice (e.g., [64]), and (3) knockout or transgenic models in which selected genes are removed or activated within the mouse. While none of these methods have directly examined the role of EGFR aberrations in ovarian cancer, some of these methods have been applied to other tumor models (e.g., glioma [65], lung adenocarcinoma [66]) in which EGFR perturbations (activating mutations) have been studied, indicating that EGFR-mediated tumor development can be successfully developed in transgenic mice.

In one study where signaling proteins downstream of EGFR induced ovarian cancer, transgenic mice harboring exogenously controllable (“floxed”) expression of phosphatase and tensin homolog (*PTEN*) and mutated K-*RAS* genes were induced to gain oncogenic K-*RAS* and lose tumor suppressing *PTEN* expression in the ovaries via injection of an adenovirus-Cre recombinase vector into the infundibulum [67]. All animals developed endometrioid adenocarcinoma of the ovary and, unlike previous ovarian tumor models, were well differentiated, reflecting similar histomorphology to human epithelial ovarian cancers. Thus, this model allows for detailed study of the endometrioid subtype of epithelial ovarian cancer at various stages of tumor development and with some manipulations could be used to study the effects of EGFR aberrations in ovarian tumor development. Mouse models for other subtypes of epithelial ovarian cancers (serous, mucinous, clear cell, transitional) await further development.

## 3. Targeting EGFR in Ovarian Cancer

While several strategies have been attempted to block EGFR activity, two types of inhibitors are currently used in the clinic: (1) monoclonal antibodies (mAbs), and (2) small molecule tyrosine kinase inhibitors (see [68, 69] for reviews). A summary of these inhibitors and their uses in clinical trials is shown in Table 2. While the various natural functions of antibodies may contribute to their utility as anticancer agents, including their role as modulators or effectors of the immune response, molecular carriers, and pharmacologic agents that directly interfere with activation of the receptor and its downstream pathways (reviewed in [70]), the focus of this paper will be on mAbs as pharmacologic agents. As indicated above by the in vitro studies in human ovarian cancer cells, EGFR and its downstream effectors may be activated directly or indirectly by numerous other signaling molecules. Since determination of which molecules are key to EGFR signaling in ovarian cancers is not completely understood, the focus will be on inhibition of EGFR and its family members.

### 3.1. Anti-EGFR Monoclonal Antibodies

Anti-EGFR mAbs that are used in the clinic typically bind to the extracellular domain of EGFR (e.g., [71, 72]). While there are potentially many different mechanisms of inhibition, in many of the known cases, the antibodies prevent ligand binding (in the case of wild-type EGFR), promote antibody-receptor complex internalization [73–75], induce transient decrease of EGFR expression [76], inhibit EGFR heterodimerization [72, 77, 78], and increase ubiquitin-mediated degradation [79]. The downstream effects of inhibition in EGFR-dependent cancer cells include decreased TGF-*α* secretion, angiogenesis, cell migration, invasion (reviewed in [80]), and induction of apoptosis [81]. Additionally, certain engineered IgG subclass antibodies in which the F_c_ region is maintained can induce antibody-dependent cell-mediated cytotoxicity or complement activation (see [82, 83] for comprehensive reviews). To reduce the likelihood of patient immune response against the therapeutic antibody, mouse mAbs have been humanized (reviewed in [84]); these are reflected by their antibody names. For example, human-mouse chimeric antibodies of 30% mouse composition are designated as “-ximab” (e.g., cetuximab); humanized antibodies with 10% mouse composition are given the “-zumab” designation (e.g., trastuzumab, matuzumab), while fully humanized antibodies are designated as “-mumab” (e.g., panitumumab).

Cetuximab (Erbitux) was the first anti-EGFR mAb tested in the clinic. Cetuximab inhibits growth of a variety of cultured cancer cells including breast, prostate, lung, colon, kidney, head and neck (reviewed in [85]), pancreas [86], and bladder [87] and can induce regression (either alone or as a combined therapy) of a number of human tumor xenografts such as epidermoid carcinoma [88], renal cell carcinoma [89], pancreatic cancer [86, 90], non-small cell lung cancer (NSCLC) [91], thyroid carcinoma [92], and glioblastoma multiforme [93]. Cetuximab demonstrates activity in patients with colorectal, head and neck, and lung cancers [94, 95].

Reports for cetuximab in ovarian cancers have appeared recently (Table 2), including its use as a single agent in a phase II trial [96] and in two other phase II trials in combination with carboplatin with or without paclitaxel (Taxol) [97, 98]. In all studies, EGFR positivity was determined by immunohistochemistry (IHC) and in two cases was used among the criteria for inclusion [96, 98]. Cetuximab therapy alone showed 4% (1/25 patients) partial response (PR) [96], while the cetuximab + carboplatin trial showed 12% (3/26 patients) complete response (CR) and 23% (6/26 patients) PR [97]. While no response rate was reported in the cetuximab + carboplatin + paclitaxel trial, progression-free survival (PFS) at 18 months was 39%, which did not meet the authors' criteria for meaningful response [98] and did not proceed to the next phase of accrual. There was no evidence of correlation between EGFR levels and patient response in any of the reports. The implications of these and subsequent results will be discussed in the “Next frontiers” section.

Among other anti-EGFR antibodies, a single multi-institution open-label phase II trial was reported in patients with ovarian cancer using matuzumab (EMD 72000) [99]. While screening for this phase II trial included EGFR positivity in the ovarian tumor as determined by IHC, no responses to therapy were observed. To date there are no approved anti-EGFR antibodies for ovarian cancer, and while there was one clinical trial involving panitumumab (Vectibix) in combination with AMG 706 and gemcitabine-cisplatin in patients with advanced cancers (including ovarian), this trial was terminated. Currently, there are no full reports of clinical trials for ovarian cancer with other anti-EGFR antibodies such as zalutumumab (HuMax-EGFr) and nimotuzumab (BIOMAb^EGFR^). Among patented mAbs directed towards EGFR that are not yet in clinical use, one has been proposed for use in ovarian cancer (patent number WO2005010151); however, as it is directed against deletion mutants of EGFR (particularly EGFRvIII), its use in ovarian cancer is likely to be limited.

Due to potential EGFR transactivation by other EGFR family members, mAbs targeting other EGFR family members have also been tested or used clinically against various cancer types such as breast and urothelial malignancies (reviewed in [100]). This includes clinical trials targeting HER2 such as a phase II multi-institutional trial in ovarian cancer in which trastuzumab (Herceptin) was used as a single agent in patients determined HER2 positive by IHC [101]. An overall response rate of 7.3% (1 CR, 2 PR) was reported. However, the relatively low frequency of *HER2* amplification in unselected ovarian cancers (e.g., 10%–23%; [35, 102]) has precluded more extensive studies. Pertuzumab (Omnitarg), a HER2 dimerization inhibitor, was administered with gemcitabine (Gemzar) in platinum-resistant ovarian cancer patients in a phase II safety study [103]; efficacy awaits further reports.

Among antibodies targeted toward other signaling molecules known to activate EGFR are monoclonals for IGF-1R, including 19D12 and EM164. These antibodies have been demonstrated to inhibit proliferation of human ovarian cancer cells [104] as well as tumor growth in mouse xenograft studies [105]. However, whether EGFR aberrations affect response to anti-IGF-1R treatment or whether inhibition can be enhanced by anti-EGFR treatment is unknown.

### 3.2. Small Molecule EGFR Inhibitors

Small molecule inhibitors, based on modeling by structure-based drug design [106] or by screening (e.g., erlotinib, [107]), appear to act intracellularly by competing with ATP binding in the catalytic region of the kinase domain, thereby abrogating enzymatic activity of the kinase and its subsequent downstream signaling effects (reviewed in [108]). Small molecule inhibitors directed against EGFR generally prevent homo- and heterodimerization between it and other EGFR family members; however, in some cases the inhibitor allows heterodimerization but prevents activation of these dimers [109]. While most mAbs are designed to target full length EGFR, many small molecule inhibitors can target mutant RTKs such as EGFRvIII that lack a critical extracellular regulatory region targeted by some of the antibodies. Small molecule inhibitors can bind reversibly (e.g., gefitinib or erlotinib) or irreversibly (e.g., CI-1033) to EGFR. The clinical significance of these different mechanisms of inhibition is not yet known.

Gefitinib (Iressa or ZD1839), which inhibits a variety of cancer cell lines and xenograft tumors (reviewed in [110]), including ovarian [111], was tested as a single agent in two trials [112, 113]. In both trials, EGFR aberrations were not included as selection criteria but were assayed via IHC for EGFR protein expression [112] or via reverse phase protein array (RPPA) for total and phospho-EGFR levels [113] as well as for *EGFR* mutations in exons 18–21 via polymerase chain reaction (PCR) amplification and nucleotide sequencing [112]. In both studies, there was no CR; 0%–4% had PR, and 4%–37% had stable disease (SD) [112, 113]. While decreased EGFR phosphorylation and expression, as determined by RPPA, was observed in >50% of gefitinib-treated patients, this was not associated with clinical benefit or response [113]. However, EGFR positivity via IHC was associated with longer PFS [112]. Additionally, a mutation in exon 19 was detected in the one partially responding patient [112], a location that was shown to be responsive to gefitinib treatment in NSCLC patients [114].

Gefitinib was also used in combination with tamoxifen in a phase II study in Germany involving patients refractory or resistant to platinum-taxane-based treatment but not prescreened for estrogen receptor or EGFR expression [115]. While this combination therapy was well tolerated, it was reported to be ineffective against platinum refractory/resistant ovarian cancer as there were no tumor responses.

Another small molecule inhibitor, erlotinib (Tarceva), demonstrated limited activity for ovarian cancer patients in a multicenter phase II trial, with only 2 chemorefractory patients in 34 demonstrating a partial response to treatment [116]. While EGFR expression was determined by IHC, low expression was not used as a criterion for exclusion. Erlotinib has also been tested in combination with other chemotherapeutic agents, including the antivascular endothelial growth factor (VEGF) antibody bevacizumab (Avastin) in a phase II trial [117], and docetaxel (Taxotere) with carboplatin in a phase Ib trial [118]. EGFR aberration or positivity was not an inclusion criterion in either study, and EGFR status was reported in only one study [117], which examined EGFR positivity via IHC and activating mutations in exons 19 and 21 via PCR amplification and sequencing. The objective response rates were 15% (2/13 patients) for the erlotinib + bevacizumab therapy [117] and 52% (12/23 patients) for erlotinib + docetaxel + carboplatin [118]. No *EGFR* mutations were detected, and one patient demonstrated EGFR positivity, but this patient was unresponsive to erlotinib + bevacizumab therapy [117]. Due to lack of improvement over bevacizumab therapy alone and two incidents of fatal gastric perforations, the erlotinib + bevacizumab study was discontinued [117]. Whether these are due to the combinatorial effects of the drugs or due to bevacizumab alone, which has been reported to induce gastric perforation [119], remains undetermined. The response rate of the erlotinib + docetaxel + carboplatin therapy was slightly lower than that of a docetaxel + carboplatin therapy previously conducted by the same group (52% versus 59%, [118, 120]), but due to good patient tolerance of the 3-drug combination, it was recommended for further studies, particularly as maintenance therapy.

Lapatinib (Tykerb, Tyverb), a dual EGFR-HER2 inhibitor [121], was tested in a multicenter phase I trial in combination with carboplatin in patients with platinum-sensitive recurrent ovarian cancer [122]. Patients were not prescreened or measured for EGFR in this study. Three of 11 patients (27%) had PR, and 3 patients (27%) had SD [122]. This treatment regimen was not recommended, as it had a low response rate and significant treatment toxicities, including grade 3–4 neutropenia and grade 4 thrombocytopenia. In addition, 2 other patients had treatment delays due to development of nondose limiting grade 3 neutropenia using the initial combination therapy regimen [122].

The irreversible pan-EGFR family inhibitor CI-1033 (Canertinib) was administered in a multicenter open-label phase II trial for ovarian cancer patients who had failed prior platinum-based therapy [123]. While baseline EGFR family levels were determined via IHC from archival patient tumor specimens, it was not used as a selection criterion. No objective response was observed, although SD was confirmed in 26%–34% of the patients (depending on the dosage). There was no association between EGFR family levels by IHC and stable disease.

Due to the relatively unremarkable results of anti-EGFR small molecules in earlier clinical trials, more recent trials have focused on small molecules that bind irreversibly or have a broader target range. For instance, BIBW2992 (Tovok) binds irreversibly to EGFR and HER2 and can inhibit both wild type EGFR and activated mutants of EGFR and HER2 [124]. BIBW2992 was shown to inhibit growth of human NSCLC cells implanted in nude mice more effectively than erlotinib [124]. Several phase I and II trials are underway with BIBW2992 as a single agent or in combination with various agents such as paclitaxel, cisplatin, or temozolomide (Temodar, Temodal) in patient groups consisting of various solid tumors including glioma, NSCLC, prostate, breast, and colorectal cancer (http://www.clinicaltrials.gov/). A few trials will screen patients for EGFR or HER2 status, whether by detection of gene amplification or by activating *EGFR* mutations. An example of a small molecule with an even broader target range is AEE788, which inhibits EGFR, HER2, and vascular endothelial growth factor receptor (VEGFR) [125]. While the current focus of AEE788 is on glioblastoma, there is also a study that assesses the safety and clinical activity of AEE788 in various solid tumors. There is currently no complete report indicating which tumor types were included, patient response, and follow up. Other small molecule EGFR family inhibitors undergoing clinical trials against solid tumors of various types (specific types not yet reported) include HKI-272 and EKB-569.

In lung cancers, sensitivity to EGFR inhibition by small molecules such as gefitinib and erlotinib is associated with *EGFR* mutation [126–129]. Therefore, Lacroix et al. analyzed *EGFR* sequences from exons 18–24 in 18 advanced epithelial ovarian carcinoma specimens from patients that displayed objective response or disease stabilization to carboplatin-paclitaxel-gefitinib treatment, along with NSCLC [130]. While 2 of 20 NSCLC samples displayed an activating deletion in exon 19 (consistent with previous reports), no *EGFR* mutations were detected in the ovarian carcinomas. However, the potential role of mutations, insertions, or deletions elsewhere in *EGFR* or other EGFR family members was not explored.

## 4. Next Frontiers in Anti-EGFR Drug Discovery

### 4.1. Improving Response to EGFR Inhibitors in Ovarian Cancer

As detailed by the list of clinical trials, the use of EGFR inhibitors as single agents or in early combination studies in ovarian cancer has met with limited success. The regimens have included EGFR-selective or less selective inhibitors and administration as single agents or in combination with other non-EGFR antineoplastic agents. One not yet widely explored possibility is whether using a combination of an externally targeting EGFR drug (i.e., mAb) with an internally targeting drug (i.e., small molecule kinase inhibitor) would produce better results. So far, there is one complete report of a phase I study that has determined optimal doses of combined cetuximab and gefitinib therapy in patients with advanced or metastatic NSCLC previously treated with platinum therapy [131]. These patients had no detectable *EGFR* amplifications or K-*RAS* mutations. The regimen, with the exception of the development of hypomagnesemia, was well tolerated. There was no objective response; however, 4 of 13 had SD. Based on these results, the group has recommended an optimum tolerated dose to use in a phase II trial.

While later studies selected patients based on EGFR positivity or overexpression via IHC, many of these trials still demonstrated low efficacy, suggesting that other methods of EGFR detection might be better suited for pre-drug screening. Quantitative approaches to assess protein level, RNA levels, gene amplification, and mutations might prove less subjective and more robust than IHC and could be included as one of the predictors of patient response. In lung cancer, gene copy number assessed by fluorescence in situ hybridization (FISH) has been reported to indicate sensitivity to EGFR inhibition (reviewed in [132]). Whether *EGFR* amplification as determined by FISH is a reliable indicator of EGFR inhibitor sensitivity for other types of cancers has not yet been conclusively assessed. Additionally, it is possible that gene increase is associated with mutational activation of EGFR, serving as a surrogate marker for mutation, and would suggest that screening by FISH might be limited to cancers in which *EGFR* is frequently mutated. At any rate, clinical trials in which better-defined measurements of EGFR status are taken into consideration have been emerging, such as screening of *EGFR* mutations in NSCLC patients prior to administration of erlotinib.

An understanding of the mechanisms leading to resistance of EGFR inhibitors could help enrich for patients likely to respond to therapy and more importantly identify rational combinatorial therapy. Resistance of tumors to anti-EGFR therapies has been discussed in a number of reviews (e.g., [133]). Furthermore, various mechanisms of chemoresistance in tumor treatment have been described (e.g., see [134, 135]). Resistance can be apparent from the onset of treatment (“intrinsic”) or develop over time (“acquired”). While resistance at the physiologic level has been attributed to mechanisms such as suboptimal immune system activity or rapid metabolism or poor absorption of the drug, resistance at the molecular level has been attributed to expression or activation of molecules or signaling pathways that can directly or indirectly override the effects of the drug (reviewed in [136]). This activation may occur via intracellular or intercellular mechanisms, and the activating intercellular source could either be another tumor cell or be the surrounding stroma (reviewed in [137]).

Anti-EGFR therapy resistance mechanisms include production of EGFR-activating ligands, receptor mutations, constitutive activation of downstream pathways, and activation of alternative signaling pathways (reviewed in [138, 139]). Another mechanism recently suggested is increased resistance to autophagic cell death upon increased EGFR expression via stabilization of the facilitated glucose transporter sodium/glucose cotransporter 1 (SGLT1) [140]. SGLT1 can transport glucose “upstream” of a glucose gradient, enabling cells to accumulate higher glucose concentrations than their environment, as in the case of cancer cells, and providing more “food” for the cell [141]. Increased SGLT1 stability is dependent on EGFR expression and not its activity [140]. Thus, agents that target EGFR activity but not its expression are likely ineffective.

Another potential mechanism of EGFR inhibitor resistance is inflammation, such as by release of the inflammatory cytokine prostaglandin E2, which in lung cancer cells induced phosphorylation of MAPK, indicating a bypass of EGFR activation (reviewed in [142]). One other consideration regarding chemoresistance is the sequence or timing of multidrug administration. Proliferation of an esophageal squamous epithelial cancer cell line possessing autocrine EGFR activity was either inhibited or enhanced depending on whether a cytotoxic drug (platinum derivative or taxane) was administered before or after an EGFR inhibitor [143]. While many of these mechanisms have been studied in other cancer types, the data for ovarian cancer is currently sparse.

Experimental results have also indicated the need to better understand the interaction of EGFR with other family members, signaling events, and the tumor environment in ovarian as well as in other cancers. As noted earlier, relative differences in levels of EGFR family members induced different dimerization partners upon stimulation by a given ligand in ovarian cancer cell lines [48]. Further, there is evidence that HER3, a family member also present in ovarian cancers and associated with increased tumor aggressiveness [144] and poor prognosis [145], plays a critical role in EGFR- and HER2-driven tumors (reviewed in [146]). Therefore, only targeting EGFR will likely be insufficient due to functional overlap by other EGFR family members. Also, in mouse studies using SU11925, a small molecule that targeted both EGFR and HER2, a higher concentration of SU11925 was required to inhibit HER2 phosphorylation in xenograft tumors than in cultured human or murine cells when relative HER2 levels in the cell were higher than EGFR [147]. These results point to a potential shortcoming of small molecule inhibitors in vivo.

As evident here and in numerous other reports on EGFR inhibitors in various cancer cell types, other signaling molecules affected by or effecting EGFR family members will have to be concomitantly examined in solid tumors. First, signaling of the EGFR family occurs primarily in *trans* with HER2 being the preferred binding partner [14]. Also, in human breast cancer cells, there is evidence that cells can escape gefitinib treatment due to increased HER3 expression induced by AKT-mediated negative feedback signaling [148]. Additionally, examining signaling proteins further downstream indicates that constitutive activation of these pathways must also be taken into consideration. For example, EGFR-overexpressing human cell lines treated with gefitinib were resistant when PTEN, the negative regulator of the PI3K/AKT pathway, was not functional [149, 150]. In NSCLC, 0 of 8 patients with both *EGFR* amplification and K-*RAS* mutation responded to erlotinib treatment compared to 4 of 5 responders with *EGFR* amplification alone [151]. Further, tumors with *RAS* mutations in several cell lineages such as NSCLC, colon, and bronchioalveolar carcinoma are resistant to anti-EGFR receptor agents and may have a worsened outcome with therapy [151–153]. This is leading to widespread testing of *RAS* mutations in patients (such as the recent study in ovarian cancer [154]) and, indeed, is approved by the European Medicines Agency as an exclusion criterion for anti-EGFR therapy in colorectal cancer in Europe. Optimal efficacy of anti-EGFR therapy is likely to require concurrent targeting of the PI3K/AKT or RAS/MAPK pathways in patients with mutational activation of these downstream components. To this end, trials that target both EGFR and the PI3K/AKT pathway have been performed or are underway, including cancers for glial cells and head and neck. While new agents that target the PI3K/AKT pathway, including XL765 or XL147, are being tested against various solid tumors in combination with erlotinib, no known combination trials exist in ovarian cancer. Also, while trials utilizing the farnesyl transferase inhibitor lonafarnib (Sarasar), which targets RAS [155], are underway, none are currently examining the combination of EGFR and RAS inhibition in any tumor type.

In addition to signaling across EGFR family members and proteins downstream, consideration of other transmembrane signaling molecules must be taken into account. Considerable data in various cell types including hepatoma [156], prostate [21], and breast [157] has shown that EGFR inhibition can be overridden by IGFR stimulation. Moreover, there is in vitro evidence in human NSCLC and head and neck squamous cell cancer cells to support therapies combining EGFR and GPCR inhibitors, such as antagonists for bradykinin (CU201) or gastrin (PD176252) (e.g., [158, 159]). Recently, amplification of the RTK gene *MET* has been shown to bypass EGFR receptor inhibition in human lung cancer cells and was present in 4 of 18 lung cancer specimens that developed resistance to gefitinib or erlotinib, supporting the idea that MET should also be targeted in EGFR-dependent cancers [160]. On the other hand, treatment of solid tumors with the dual EGFR-VEGFR inhibitor vandatanib (ZD6474 or Zactima) was ineffective [161]. Based on these reports and the emergence of numerous potential EGFR-mediated signaling proteins of interest in ovarian cancers, determination of which proteins play crucial roles in ovarian tumors might prove to be a challenging process. High-throughput methods such as gene expression arrays and RPPA should help in determining which genes and proteins are modulated upon single and combination treatment of ovarian cancer cell lines and tissues. For example, Skvortsov et al. have used 2-dimensional gel electrophoresis and mass spectrometry to identify proteins associated with sensitivity or resistance to C225 in two colon cancer cell lines [162]. Additionally, development of robust algorithms to predict effective drug combinations (e.g., [163]) should aid in streamlining high-throughput studies and increase the likelihood of finding successful combinations.

Despite these challenges, reports utilizing adherent human epithelial cancer cell lines and tumor types suggest that mechanisms of resistance and methods to overcome resistance could be determined and incorporated into ovarian cancer therapies. For instance, MAPK phosphorylation was not inhibited in an EGFR-positive, gefitinib-resistant human bladder cancer cell line upon gefitinib treatment, while MAPK phosphorylation decreased in an EGFR-positive, gefitinib-sensitive cell line [164]. Moreover, in the gefitinib-sensitive cell line, increased GSK-3*β* activity and decreased cyclin D1 levels were observed upon gefitinib treatment and correlated with responsiveness. Additionally, platelet-derived growth factor receptor-*β* (PDGFR-*β*) was observed to short circuit the EGFR/MAPK pathway in the gefitinib-resistant cells [164]. These results suggest that, in bladder cancer, MAPK kinase phosphorylation could be a marker for resistance while GSK-3*β* activation or cyclin D1 levels could be a marker for sensitivity of EGFR drug treatment, and that inhibition of both EGFR and PDGFR-*β* would be more effective in treatment of EGFR-positive bladder cancers than EGFR alone.

### 4.2. Improving Understanding of EGFR Processes in Ovarian Cancer

With the emergence of high-throughput technologies and their accompanying development and refinement of data analyses, reports contributing to further understanding of ovarian cancers have emerged. Among the first reports utilizing gene arrays was that of Wang et al., who identified genetic differences between human ovarian tumor specimens (comprising 5 different histopathologic types) and normal ovarian tissue [165]. Later studies expanded the number and refined the analyses of histopathologic types of samples (serous papillary, clear cell, endometrioid, undifferentiated, and adenocarcinomas) included in the analyses (e.g., [166]), as well as compared drug (primarily platinum) sensitive and resistant samples [167]. While the number of samples analyzed in depth is increasing, this number is still relatively small; whether the profile of EGFR-positive ovarian cancers is different from that of other prominent molecular markers is unknown. Moreover, the most comprehensive profiles characterized thus far have focused on gene alterations, via comparative genomic hybridization or gene microarrays (reviewed in [168, 169]), which provide an incomplete profile of ovarian cancer cells, particularly in the case of protein signaling-dependent alterations such as EGFR activation. Thus, more information derived from proteomic studies is needed.

Based on the current outcomes of EGFR targeted therapy in ovarian cancers, it is evident that patients should be screened for EGFR status including amplification and mutation; additionally, screening for other EGFR family members and key downstream effector proteins such as RAS and PTEN would be preferable. Also, while *EGFR* in ovarian cancers has been screened for potential activating events via presence of *EGFRvIII* [38, 39] or activating mutations in the kinase domain [6, 35–37], it is possible that ovarian cancers might have a yet unidentified *EGFR* activating “hot spot.” Screening and analysis of full-length *EGFR* will be required to determine if this is the case.

Determination of other molecular markers for likely responders or nonresponders toward anti-EGFR therapies should also be performed; identification of such markers could be facilitated by high-throughput methods that can be correlated with patient response. High-throughput methods could also be used to aid in developing predictive models of drug combination in patients, such as by testing well-defined chemotherapeutic drugs in a large number of cancer cell lines and performing cell “population studies,” to better correlate drug response with precisely defined oncogene status (e.g., specific mutations, gene amplification), such as with EGFR [170]. Further studies of other proteins affecting or affected by EGFR activity, some of which have been discussed above, should also be performed to clarify their roles in ovarian cancer, both independently and in context with EGFR activation. Further, the role of EGFR in different ovarian cancer histotypes should be examined. Additionally, preclinical combination therapy reports such as by Morelli et al. [143] suggest that more studies should be performed on determining proper scheduling of multiple therapies as well as examination of previously untested drug combinations. Also of great benefit is designing more streamlined and rational methods for performing drug combination studies, such as by development of search algorithms to determine optimal doses of combined drugs [171].

## 5. Conclusion

EGFR and its family members play a variety of roles in oncogenesis and tumor progression in different cancer and cell types. To date, clinical studies using EGFR antagonists in ovarian cancer have shown limited efficacy. As we learn more about the complexities of specific signaling changes associated with EGFR mutation and overexpression, future studies using EGFR antagonists in ovarian cancer should focus on determining reliable predictors for patient responsiveness to anti-EGFR therapy such as by obtaining good biomarker profiles and utilizing assays most appropriate to determine EGFR status as well as developing rational combination therapies with EGFR inhibitors. These determinations should be facilitated by the use of high-throughput methods, as well as development of robust algorithms to help design experiments and analyze results. Continuing these studies in ovarian and other types of cancers will increase our likelihood of achieving success in targeting EGFR-dependent tumors.

## Figures and Tables

**Table 1:** *Summary of assays used in detecting EGFR in vitro and in vivo*. Aside from high-throughput methods (such as cDNA arrays, comparative genomic hybridization, and reverse phase protein arrays) and xenograft tumor assays, more broadly encompassing biological methods such as assays for invasion, migration, or gene knockouts have been excluded. cDNA: complementary DNA; PCR: polymerase chain reaction.

EGFR assay method	Assay output	Performed in ovarian cancer?	Platform for ovarian cancer	References for ovarian cancer
cDNA Array	Detection of mRNA levels of various genes	Yes*	Patient tissue, Human cell lines	[172]
Comparative Genomic Hybridization	Detection of copy number changes in chromosomes	Yes*	Patient tissue, Human cell lines	[173]
Chromatin Immunoprecipitation	Detection of stable protein-DNA associations	No		
Coimmunoprecipitation + Western blotting	Detection of stable protein-protein associations	No		
Crystallography	Determination of entire structure or portions of molecule; interacting molecules	No		
Enzyme-linked Immunosorbent Assay	Determination of amount of protein in sample	Yes	Patient tissue	[174]
Fluorescence/ Chromogenic in situ Hybridization	Determination of gene copy number	Yes	Patient tissue	[3, 6, 35, 36]
Flow Cytometry/ Fluorescence-Activated Cell Sorting	Determination of protein levels at cell surface	Yes	Patient tissue, Human cell lines	[175–179]
Immuno-histochemistry/ Immunocyto-chemistry/ Immunofluorescence (includes Tissue Microarrays)	Determination of presence, location, or amount of protein in tissue/cell	Yes	Patient tissue, Patient effusions, Human cell lines	[4, 5, 35–37, 40, 41, 43, 46, 97, 117, 123, 178, 180–195]
In vitro Kinase Assay	Measurement of intrinsic kinase activity	No		
Mass Spectrometry after Protein Enrichment /Purification (e.g., Immunoprecipitation, Chromatographic Separation, Baculovirus Expression)	Detection of protein modification sites (e.g., phosphorylation, glycosylation); changes in protein levels or proteomic profiles, protein-protein complexes	No		
Microscopic Techniques (e.g., Confocal)	Determination of presence, location, or amount of protein in cell	No		
Mulitplex Antibody Arrays (Solid Phase or Bead Based)	Detection of multiple molecules (usually proteins) of interest	Yes*	Patient serum, Human cell lines	[196, 197]
Northern Blotting	Determination of steady-state RNA levels	Yes	Patient tissue, Human cell lines	[43, 186, 193, 198, 199]
PCR + DNA analysis (e.g., Sequencing, Restriction Fragment Length Polymorphisms, Denaturing Gradient Gel Electrophoresis)	Detection of known mutations/ polymorphisms	Yes	Patient tissue, Human cell lines	[6, 35–37, 117, 130, 187, 200]
Quantitative PCR	Measurement of RNA levels of interest	Yes	Human cell lines	[39, 174, 201]
Radioligand Binding/ Radioimmunoassay	Estimation of number of receptors; determination of ligand or agonist/ antagonist binding kinetics	Yes	Patient tissue, Patient effusions, Human cell lines	[42–45, 199]
Reverse Phase Protein Array	Determination of levels of several proteins and protein modifications of interest	Yes	Patient tissue, Patient effusions	[202, 203]
Reverse Transcription-PCR + Southern Blotting	Determination of mRNA levels	Yes	Human cell lines, Rat cell lines	[198, 204]
Southern Blotting	Detection of gene of interest	Yes	Rat cell lines	[198]
Tryptic Digests + Peptide Resolution (e.g., Reverse Phase High Performance Liquid Chromatography)	Determination of phosphorylation sites of protein	No		
Western Blotting	Determination of protein abundance, protein-associated modifications (e.g., phosphorylation, cleavage, ubiquitination)	Yes	Patient tissue, Human cell lines	[38, 39, 46, 48–50, 56, 147, 175, 177, 178, 181, 186, 196, 200, 201, 204–212]
Xenograft Tumors	Determination of effect of gene/cell perturbation on tumor growth	Yes	Human and mouse cell lines	[47, 49, 147, 178, 213–219]

*EGFR was detected and reported, but samples were not necessarily preselected for alteration of *EGFR* sequence, expression, or activity.

**Table:** (a) Monoclonal Antibodies

Study and Year	CT no.	Phase	# Pts	Therapy	Selection criteria	Outcome	Comments
Secord et al. 2008 [97]	NCT 00086892	II	28	Cetuximab + Carboplatin	Recurrent, platinum-sensitive disease	CR: 3 pts	Response rate criteria not met for next stage of accrual. 26 pts were EGFR positive by IHC.
						PR: 6 pts	
						SD: 8 pts	
Konner et al. 2008 [98]	NCT 00063401	II	40	Cetuximab + Paclitaxel + Carboplatin	Grade III-IV debulked tumor, EGFR positive by IHC	Median PFS: 14.4 months	Combination was adequately tolerated. No increase in PFS when compared to historical data.
						PFS at 18 months: 39%	
Schilder et al. 2009 [96]		II	25	Cetuximab	Persistent or recurrent ovarian or primary peritoneal disease, EGFR positive tumors by IHC		12 serologic markers examined before and during treatment. No correlation between PFS and marker changes, but high baseline of markers associated with earlier disease progression.
						PR: 1 pt	
						SD: 9 pts	
	
Seiden et al. 2007 [99]	NCT 00073541	II	37	Matuzumab	Recurrent platinum-refractory disease, EGFR positivity by IHC	No objective response	Primary objective was pharmacodynamic; signal transduction evaluation. 75 pts were screened for EGFR status.
						SD: 16%–22%	
Bookman et al. 2003 [101]	GOG-160	II	41	Trastuzumab	Persistent and/or refractory disease with 2-3+ HER2 by IHC	CR: 1 pt	Serum HER2 levels not associated with clinical outcome.
						PR: 2 pts	

**Table:** (b) Small Molecule Inhibitors

Study and Year	CT no.	Phase	# Pts	Therapy	Selection criteria	Outcome	Comments
Posadas et al. 2007 [203]	NCT 00049556	II	24	Gefitinib	Platinum-refractory disease	No objective response	Protein correlates done with RPPA. No significant correlation between EGFR phosphorylation and tumor response
						SD: 37% for >2 months	
Schilder et al. 2005 [112]	NCT 00023699	II	27	Gefitinib	Persistent or recurrent disease	PR: 1 pt	Analyses suggest trend towards responsiveness in EGFR positive (by IHC) pts. Activating mutations documented in the PR pt.
Wagner et al. 2007 [115]	NCT 00189358	II	56	Gefitinib + Tamoxifen	Disease refractory or resistant to platinum-taxane-based therapy	No objective response	EGFR positivity not a prerequisite; EGFR status not determined
						SD: 16 pts	
Gordon et al. 2005 [116]		II	34	Erlotinib	Relapsed or progressive disease, EGFR positivity by IHC	PR: 2 pts	Primary goal was to estimate the objective tumor response rate to erlotinib as a single agent.
						SD: 15 pts	
	
Vasey et al. 2008 [118]		Ib	45	Erlotinib + Docetaxel + Carboplatin	Chemonaïve pts	CR: 5 pts	Phase Ib dose finding study. Addition of erlotinib to other agents did not increase response rate.
						PR: 7 pts	
						(23 evaluable)	
Nimeiri et al. 2008 [117]	NCT 00126542	II	13	Erlotinib + Bevacizumab	Recurrent or refractory disease, ≤2 prior cytotoxic chemotherapies; no previous anti-EGFR or VEGFR therapies		No indication of improvement over bevacizumab treatment only. No *EGFR* mutations detected; one EGFR 2+ IHC staining detected.
						CR: 1 pt	
						PR: 1 pt	

Kimball et al. 2008 [122]	NCT 00317434	I	11	Lapatinib + Carboplatin	Recurrent, platinum-sensitive disease	PR: 3 pts	No screening or measurement of EGFR or HER2 performed.
						SD: 3 pts	
Campos et al. 2005 [123]		II	105	CI-1033	Relapsed or refractory disease	No objective response	Baseline HER1-2 levels determined by IHC. No association between HER levels and SD.
						SD: 26–34%	
